# A narrative review of school-based screening tools for dyslexia among students

**DOI:** 10.3389/fpubh.2025.1654470

**Published:** 2025-10-23

**Authors:** Rinad Bakhti, Nishani Fonseka, Federica Amati, Dasha Elizabeth Nicholls, Dougal Hargreaves, Antonio Lazzarino, Lucy McCann, Sara-Nicole Gardner, Krishan Narayan, Helen Kerslake, Alex Weston, Shamini Gnani

**Affiliations:** 1 Department of Brain Sciences, Faculty of Medicine, Imperial College London, London, United Kingdom; 2 Keele University, Keele, United Kingdom; 3 School of Public Health, Faculty of Medicine, Imperial College London, London, United Kingdom; 4 Wolfson Institute of Population Health, Queen Mary University of London, London, United Kingdom; 5 Institute of Psychiatry, Psychology and Neuroscience, King's College London, London, United Kingdom; 6 Royal Borough of Kensington and Chelsea, London, United Kingdom; 7 Listen to Act, London, United Kingdom

**Keywords:** school-based screening tools, learning disability, dyslexia screening, sensitivity and specificity, systematic review

## Abstract

**Background:**

Early detection and intervention of dyslexia in children and young people (CYP) can help mitigate its negative impacts. Schools play a crucial role as a key point of contact for dyslexia screening.

**Objective:**

In this review, we examined the range of screening tools and reported sensitivities and specificities in school settings to identify CYP with dyslexia and explored variations in how tools captured the socio-demographic characteristics of screened student's groups.

**Design:**

Narrative review.

**Methods:**

We searched five electronic databases: EMBASE, MEDLINE, PsychInfo, Cochrane, and Scopus (2010–2023) to identify worldwide school-based dyslexia screening studies conducted in CYP aged 4–16 years. Three independent researchers screened the papers, and data were extracted on the sensitivity and specificity of the screening tools, the informants involved, the prevalence of dyslexia among those who screened positive, and the socio-demographic characteristics of the identified CYP.

**Results:**

Sixteen of 6,041 articles met the eligibility criteria. The study population ranged from 95 to 9,964 participants. We identified 17 different types of school-based dyslexia screening tools. Most studies combined screening tools (mean number of 3.7, standard deviation = 2.7) concurrently to identify dyslexia. Three studies used a staged approach of two and three stages. Developmental Dyslexia and Dysorthographia and Raven Progressive Matrices were the most used tools. The percentage of cases screening positive for dyslexia ranged from 3.1 to 33.0%. Among CYP identified by screening with dyslexia, there were missing socio-demographic data on gender (50%) and socio-economic status (81%) and none on ethnicity.

**Conclusion:**

A variety of screening tools are used to identify children and young people (CYP) with dyslexia in school settings. However, it is unclear whether this wide range of tools is necessary or reflects variations in definitions. Greater collaboration between researchers and front-line educators could help establish a solid evidence base for screening and reduce the inconsistencies in approach. In the meantime, a practical and beneficial approach may involve starting with a highly sensitive screening tool, followed by more specific tests to assess detailed deficits and their impact.

## Introduction

Dyslexia is widely recognized as a learning disorder and neurodiverse condition that affects the accuracy and fluency of reading in ways that are atypical relative to an individual's age, education, and/or intellectual ability (1, 2). A child's phonological processing ability is considered one of the strongest predictors of literacy acquisition (3, 4). However, definitions of dyslexia vary across disciplines, regions, and contexts. For example, some frameworks view dyslexia as a distinct diagnostic disorder, while others treat it as a cluster of reading-related difficulties or symptoms. Although there is ongoing debate and variation in how dyslexia is defined across disciplines and contexts, we adopt the definition of dyslexia as a neurodevelopmental condition primarily characterized by difficulties with accurate and/or fluent word recognition, along with poor spelling and decoding abilities. This aligns with definitions provided by the Rose Report (5) and Snowling and Melby-Lervåg (6). Dyslexia may co-occur with other learning or emotional difficulties. It is one of the most prevalent neurodiverse conditions and commonly occurs alongside conditions such as dyspraxia, dyscalculia, Attention Deficit Hyperactivity Disorder (ADHD), and Autism Spectrum Disorder (ASD) (7).

Although global dyslexia prevalence among children and young people (CYP) is estimated to be 7.1% (8), figures can vary widely depending on criteria for diagnosis and language (9, 10). The long-term effects of dyslexia for CYP compared to those without the condition include social, emotional, and behavioral issues, school exclusion, increased likelihood of attending a youth offending institute, and overall poor educational outcomes (11–13).

Schools are an ideal setting to universally screen for children with dyslexia and mitigate against harmful educational outcomes. Yet children with dyslexia are often diagnosed late due to a lack of awareness among teachers and parents regarding the signs and symptoms of the condition. Adopting a universal approach to screening in schools (screening tools can be used before school entry) (14, 15) can help overcome barriers that exist in identifying children with dyslexia that may relate to finance and other socio-demographic factors.

The precision of a school-based screening tool for dyslexia is important and should ideally result in a high percentage of true positives (children who are correctly identified as at risk, known as its sensitivity) and a high percentage of true negatives (children who are correctly identified as not at risk, known as its specificity (16)). However, no single screening tool or set of screening tools can achieve 100% sensitivity and specificity, thus, rendering false positives and false negatives inherent to any tool (3) and is a trade-off and balance between sensitivity and specificity (16, 17). Additionally, screening methods for dyslexia in school settings can be broadly categorized into subjective (e.g., teacher or parent questionnaires, self-report tools) and objective methods (e.g., direct testing of phonological awareness, rapid automatized naming, or decoding skills). Each category carries its own strengths and limitations in terms of reliability, feasibility, and alignment with diagnostic criteria.

Currently, many schools use different approaches to screen for CYP with dyslexia. This review was guided by answering the following research questions: (1) What dyslexia screening tools are most commonly used in school-based settings for children and young people aged 4–16 years? (2) How frequently are combinations of tools employed, and in what ways? (3) Which tools demonstrate the highest reported sensitivity and specificity? and (4) What gaps remain in the evidence regarding socio-demographic equity of school-based screening?

## Methods

### Context

Ethical approval was not sought for this study as it was an evidence synthesis of existing published research.

### Patient and public involvement

The topic for this review was identified in consultation with a young people's advisory group in Northwest London, United Kingdom (https://www.arc-nwl.nihr.ac.uk/research/multimorbidity-and-mental-health/arc-outreach-alliance/young-peoples-advisory-group-ypag). The advisory group were first involved in the design of the study and advised on the neurodiverse conditions to study.

### Search strategy

#### Information sources

We electronically searched five academic databases: EMBASE, MEDLINE, PsychInfo, Cochrane, and Scopus in September 2022. The reference list of all included articles was screened for additional studies. An updated search was conducted in February 2024, which yielded one further article, and again in August 2024, which did not identify any additional articles. Search terms are available in Supplementary File 1.

Keywords and index terms from relevant articles were identified as part of an initial search of MEDLINE and PSYCHINFO and helped inform the development of a full search strategy. The terms were mapped using a PICO (population, intervention, control, and outcome) approach and cross-checked with clinical and academic colleagues. Further advice was sought from a medical librarian to ensure relevant terms were included. The search strategy was adapted for each database to reflect both school-based screening and dyslexia-related symptoms. As a narrative review, we did not perform a formal risk-of-bias assessment.

### Eligibility criteria

We excluded all studies that were published before 2010 as research in how neurodiverse conditions are perceived has changed significantly. Supplementary Table 1 outlines the eligibility criteria. We excluded children under 4 years and over 16 years, as access to full time education is not a requirement in many countries. We screened for articles that had dyslexia as a condition. Our inclusion criteria focused on screening tools used in real-world school settings, as our aim was to assess tools most commonly used in educational settings. Tools such as Test of Word Reading Efficiency (TOWRE) and York Assessment of Reading for Comprehension (YARC), while highly regarded in diagnostic contexts, were excluded unless explicitly used as part of school-wide screening initiatives, as our focus was on screening tools feasibly implemented at scale in educational settings. We excluded gray literature, defined here as unpublished theses, internal school evaluation reports, and non-peer-reviewed conference abstracts, to maintain a focus on peer-reviewed research.

We included studies that evaluated tools used in school settings to screen for dyslexia-related symptoms (e.g., phonological deficits, decoding difficulties) rather than tools designed solely for diagnostic purposes. Studies involving populations with concurrent conditions (e.g., ADHD) were included if the screening tool aimed to detect dyslexia symptoms independently. Although not designed to detect dyslexia, tools assessing emotional health (e.g., SDQ) or cognitive ability (e.g., Raven's Matrices) were also included when they were integrated into a school-based dyslexia screening protocol.

### Study selection

Following the search, we collated all identified citations and uploaded these into Covidence systematic review software (18). Duplicates were removed. Titles and abstracts were screened by three independent reviewers for assessment against the review's eligibility criteria. The full text of selected citations was assessed by three independent reviewers. Reasons for exclusion of sources of evidence in full text were recorded. Any disagreements that arose between the reviewers at each stage of the selection process were resolved through discussion with the wider research team.

### Data collection process and data items

Data were extracted by two independent reviewers into an Excel spreadsheet. We reported on sensitivity and specificity of the screening tool, its informants (e.g., teachers, parents, and self-report), associated prevalence of screen positive cases of dyslexia, and socio-demographic characteristics. Any disagreements between the reviewers were resolved through discussion, and with additional research authors. Additional or missing data were not sought from authors of papers included.

### Types of sources

We included experimental and quasi-experimental study designs (randomized controlled trials, non-randomized controlled trials, before and after studies, and interrupted time-series studies) and analytical observational studies (prospective and retrospective cohort studies, case-control studies, and analytical cross-sectional studies). Case studies and opinion papers and non-school-based screening studies were excluded from this review.

## Results

**Figure 1:**
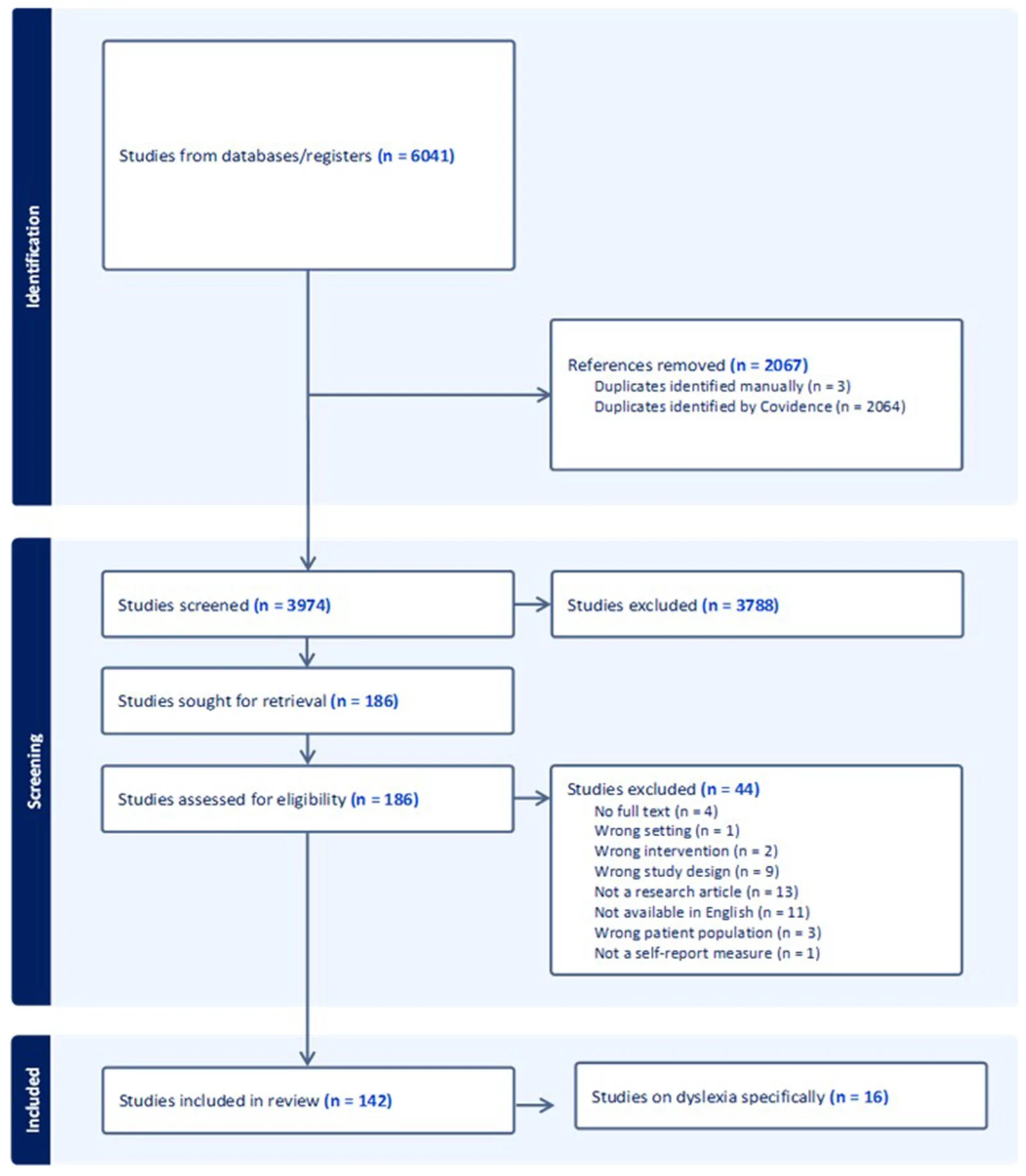
PRISMA flow diagram of study selection.

Figure 1 presents the Preferred Reporting Items for Systematic Reviews and Meta-Analyses (PRISMA) diagram and details the search and selection process applied during the screening of articles.

We identified 16 eligible studies of school-based screening for dyslexia in this review. Table 1 presents a summary of these studies including screening tools used and study outcomes (their informants, sensitivity, specificity, the language of administration and prevalence of CYP screening positive for dyslexia by gender and demographics) to improve transparency and facilitate comparison across different contexts. Studies were conducted across 11 countries and six World Health Organization regions (19): European (*N* = 5), Western Pacific (*N* = 4), the Americas (*N* = 2), Eastern Mediterranean (*N* = 2), Southeast Asia (*N* = 2), and African (*N* = 1). Screening tools were available in different languages including English, Mandarin, and Italian.

**Table 1:** Summary of included studies of screening tools used in identifying Children and Young People with dyslexia in school settings (*N* = 16).

**Study number**	**Author (publication year)**	**Country**	**Sample size**	**Gender distribution (girls, boys)**	**Age group (Years)**	**Screening tool used**	**Prevalence of screening positive for dyslexia (%); screening positive by gender; sensitivity and specificity of tool**
1	Ashraf and Majeed (27)	Pakistan	500	250 girls; 250 boys	11–17	- Bangor Dyslexia Test - Raven's Progressive Matrices - Slosson Intelligence Tests	5.4%; 4.4% girls; 6.3% boys; NA
2	Ashraf and Najam (28)	Pakistan	666	282 girls; 384 boys	11–17	Learning Disabilities Checklist	33.0%; 13.7% girls; 20.0% boys; NA
3	Barbiero et al. (25)	Italy	1,357	663 girls; 702 boys	8–10	Screening stage 1: - RSR-DSA derived questionnaire (Questionario per la rilevazione di difficolta‘ e disturbi dell'apprendimento) - BVSCO: (writing skills, dictation task) Screening stage 2: - Developmental Dyslexia and Dysorthographia 2nd edition (DDE-2): (word and non-word reading subtest) - Wechsler Intelligence Scale for Children 3rd edition (WISC-III): vocabulary and block design subtest Screening stage 3: - Raven's Progressive Matrices - MT battery (Prove di lettura MT per la scuola elementare-2) - DDE-2: (speed and accuracy of word and non-word reading and accuracy of spelling subtest) - Strengths and Difficulties Questionnaire	3.1% (diagnosis); NA; NA
4	Barbiero et al. (26)	Italy	9,964	4,679 girls; 4,864 boys	8–10	Screening stage 1: - RSR-DSA derived Questionnaire - BVSCO: (dictation test) Screening stage 2: - DDE-2: (word and non-word reading subtest) - WISC-III: (vocabulary and block design subtests) Screening stage 3: - Raven's Progressive Matrices - MT battery - DDE-2: (speed and accuracy of word/non-word reading and accuracy of spelling subtest) - Strengths and Difficulties Questionnaire	3.5% (diagnosis); 1.4% girls, 1.9% boys; NA
5	Bassôa et al. (39)	Brazil	95	50 girls; 45 boys	NA (mean *=* 9.3)	Screener for Reading and Writing instrument	9.0%; NA; NA
6	Cai et al. (20)	China	1661	779 girls 882 boys	NA (mean *=* 9.2)	- Chinese Character Recognition Measure and Assessment Scale - Pupil Rating Scale Revised Screening for Learning Disability - Strengths and Difficulties Questionnaire [Chinese] - Combined Raven Test	4.9 %; 1.9% girls, 7.5% boys; NA
7	Chan et al. (30)	China	1,063	405 girls; 658 boys	6–14	- Hong Kong Behavior Checklist of Specific Learning Difficulties in Reading and Writing (HKBCL) - Hong Kong Test of Specific Learning Difficulties in Reading and Writing	NA; NA; HKBCL (86% sensitivity; 33% specificity)
8	Ching et al. (31)	China	947	475 girls; 472 boys	11–15	Hong Kong Specific Learning Difficulties Behavior Checklist for Junior and Secondary students	NA; NA; 86% sensitivity; 81% specificity
9	Choudhary et al. (21)	India	468	NA	7–11	Screening stage 1 - Dyslexia Assessment Questionnaire Screening stage 2/Diagnosis - Dyslexia Screening Tests Junior	17.5% (stage 1), 10.3% (combined diagnosis); 7.4% girls; 11.4% boys; NA
10	ElSheikh et al. (40)	Egypt	567	262 girls; 305 boys	9–12	- Kiddie Schedule for Affective Disorders and Schizophrenia - Reading Disability Test	11.3%; 4.8% girls, 6.5% boys; NA
11	Fletcher et al. (23)	USA	945	NA^*^	NA^*^	- Comprehensive Test of Phonological Processes - Rapid Automatized Naming - Alphabetic knowledge - Peabody Picture Vocabulary Test-Revised - Word reading - Woodcock Johnson–Revised: (letter-word identification, word attack/pseudowords and passage comprehension subtest) - The Primary Reading Inventory Scale development	NA; NA; combined tests 89% sensitivity; 67% specificity.
12	Lerthattasilp et al. (22)	Thailand	1,017	502 girls; 515 boys	NA (mean *=* 7.0)	- Raven's Progressive Matrices - A reading ability test	15.8%; 8.8% girls, 22.9% boys; NA
13	Nergård-Nilssen and Friborg (32)	Norway	1,100	NA^*^	6–12	Dyslexia Marker Test for Children	NA; NA; Sensitivity: 90.6%; Specificity: 70%
14	Poulsen et al. (41)	Denmark	164	77 girls; 87 boys	NA (mean *=* 6.1)	- Rapid automatized naming - Letter knowledge - Phonemic awareness - Paired associate learning - Grade 0 oral word reading accuracy - Grade 1 and 2 oral words reading accuracy - Grade 1 and 2 oral words reading fluency	NA; NA; - Grade 0 screening: 80% sensitivity and 29% specificity - Grade 1 screening: 80% sensitivity and 7% specificity
15	Snowling et al. (24)	UK	146	42 girls; 104 boys	6	- York Assessment of Reading for Comprehension (YARC letter sound knowledge; early word reading subtest; sound deletion and isolation subtest) - Clinical Evaluation of Language Fundamentals 3rd edition: - Rapid Automatic Naming (colors subtest) - Working Memory Test Battery for Children (Digit recall forward and listening recall) - Wechsler Intelligence Scale for Children 3rd edition (Block design/non-verbal subtest) - Teacher ratings of ‘phonic phases'	16.4%; NA; NA
16	Sun et al. (42)	China	5,063	2,482 girls; 2,581 boys	4–11	- Dyslexia Checklist for Chinese Children - Pupil Rating Scale Revised Screening for Learning Disabilities - Chinese language test - Combined Raven's test	3.9%; 1% girls, 2.9% boys; NA

NA denotes not available.

^*^Authors report that genders are “roughly equally represented”.

Participants ranged in age from 4 to 16 years, and the study sample size ranged from 95 to 9,964 participants. In general, early primary school studies tended to emphasize phonological awareness and letter knowledge, whereas studies in older primary and secondary school students focused more on fluency, comprehension, and spelling. This suggests that the applicability of a tool may be age- or stage-dependent.

Seven studies recruited over 1,000 participants: China (*N* = 3), Italy (*N* = 2), Norway (*N* = 1) and Thailand (*N* = 1) and of these four studies reported similar screening positive prevalence for dyslexia; 3.1–4.9%. Of the eight studies which included a gender distribution for dyslexia screening prevalence, all of them reported higher figures among boys compared to girls.

Of the 16 studies we identified in our review, seven studies reported the socio-economic status of CYP screened. Of these seven, only three studies reported on the socioeconomic status of participants and its association with screening positive for dyslexia (20–22). Socio-economic status was assessed using parental occupation and education, and monthly family income, alone or in combination. Cai et al. (20) found that high levels of parental education was associated with less likelihood of screening positive for dyslexia while no significant association was found between family income and screening positive for dyslexia. Similarly, Lerthattasilp et al. (22) found higher monthly incomes were associated with less likelihood of screening positive for dyslexia and an association between parents with low levels of education screening positive for dyslexia. However, an earlier study by Choudhary et al. (21) showed that the socioeconomic status between CYP with dyslexia and those without (control group) did not differ. Socio-demographic factors such as parental education and family income were inconsistently reported, limiting conclusions regarding equity and generalizability.

No studies reported on the ethnicity of CYP who screened positive for dyslexia and only one study conducted in the USA (23) reported on ethnicity of the study population's CYP reflecting limited socio-demographic reporting. Other studies reported ethnicity in terms of nationality such as China or made no mention of ethnic status.

Dyslexia screening tools were used in combination and concurrently in 12 studies; the mean number of tools used was 3.7 (standard deviation = 2.7), with four studies using only one tool and two studies using eight tools to screen, reflecting variability in methodological approaches. Three studies adopted a staged screening for dyslexia whereby further screening tests were administered if a certain cut-off was achieved at the first stage. Different combinations of dyslexia screening tools were used as well as ‘other tools' which assessed the impact of dyslexia in terms of function as well as other concurrent problems. For example, the strengths and difficulties questionnaire (20) which is an emotional and behavioral screening tool and the Wechsler intelligence scale for children (24). Supplementary Table 2 shows the other screening tools that were identified in this review.

The most used dyslexia-specific screening tool was the Developmental Dyslexia and Dysorthographia 2nd edition (DDE-2). This albeit different subtests of DDE-2 were used four times in two separate studies (25, 26). Among other tools, the most used was the Raven Progressive Matrices which was used in four studies (22, 25–27).

Five studies did not report the prevalence of screening positive for dyslexia highlighting gaps in reporting and limiting cross-study comparisons. Among the remaining 11 studies the reported figure ranged from 3.1% to 33%. The highest figure of 33% was observed in a study from Pakistan (28) where the authors used the Learning Disability Checklist, a non-specific dyslexia test, which may explain the high figure and the likelihood of false positive. High rates of prevalence may be attributed in part to the lack of early assessment and screening strategies at school level (28, 29). In general, reported difficulties in using these tools included time constraints, need for trained personnel, and variability in teacher familiarity with the measures.

Additionally, 11 studies did not report the sensitivity or specificity of the screening tools used. In the five studies which did report these metrics, sensitivity was consistently higher than specificity in all combination of tools administered, for example, Chan et al. (30) reported the sensitivity of the Hong Kong Behavior Checklist of Specific Learning Difficulties as 86% while the specificity was 33%. Nergård-Nilssen and Friborg (32) reported the highest sensitivity of their screening protocol (90.6%) which used the Dyslexia Marker Test for Children. Fletcher et al. (23)'s combined seven tools to achieve the second highest sensitivity metric of 89% screening children approximately aged 5 years. It included the following: (1) Comprehensive Test of Phonological Processes, (2) Rapid Automatized Naming, (3) Alphabetic knowledge, (4) Peabody Picture Vocabulary Test-Revised, (5) Word reading, (6) Woodcock Johnson–Revised (WJR), and (7) Primary Reading Inventory Scale development.

The screening tool with the highest reported specificity of 81% was in a study conducted by Ching et al. (31) where students were screened using the Hong Kong Specific Learning Difficulties Behavior Checklist for Junior and Secondary. The Dyslexia Marker Test for Children in a study by Nergård-Nilssen and Friborg (32) scored the second highest of 70%.

We identified 17 different dyslexia school-based screening tools that were used in the review (Table 2). Students were the main informant (*N* = 11) followed by teachers (*N* = 5) and then one tool used by parents (Dyslexia Assessment Questionnaire (21)).

**Table 2:** Summary of screening tools used for dyslexia and included in studies identified by the review (*N* = 17).

**Study number**	**Name of screening tool**	**Description of screening tool**	**Number of items/subtests**	**Informant**
1	Bangor Dyslexia Test	Focusing on verbal and phonological processing	10 items	Student
2	Chinese Character Recognition Measure and Assessment Scale	For Mandarin-speaking children. Students write combined words with presented Chinese characters in a limited time. Literacy ability computed according to accuracy rate of combined words	NA	Student
3	Developmental Dyslexia and Dysorthographia 2nd edition (DDE-2)	Assess reading speed and accuracy (number of errors) in reading word lists (4 lists of 24 words) and non-word lists (3 lists of 16 non-words)	144 items	Student
4	Dyslexia Assessment Questionnaire	Questions on child's difficulties in spelling or reading and family history of difficulties	20 items	Parents
5	Dyslexia Marker Test for Children	Subtests include letter knowledge test, phoneme isolation, phoneme deletion, rapid automatized naming, working memory, decoding and spelling	6 subtests	Student
6	Dyslexia Screening Tests Junior	Subtests include rapid naming, phonemic segmentation, writing verbal fluency rhythm and vocabulary.	NA	Student
7	Hong Kong Behavior Checklist of Specific Learning Difficulties in Reading and Writing	Checklist of student reading-related behavioral characteristics	45 items	Teacher
8	Hong Kong Test of Specific Learning Difficulties in Reading and Writing	Subtests include reading, dictation/copying, writing, general performance, mathematics, language, memory, concentration, sequencing ability, motor coordination, spatial orientation and social emotional adjustment	12 subtests	Student
9	Hong Kong Specific Learning Difficulties Behavior Checklist Junior and Secondary students	Checklist of student reading-related behavioral characteristics	52 items	Teacher
10	Learning Disabilities Checklist	35 items measuring dyslexia, dysgraphia, dyscalculia on a dichotomous scale	35 items	Student
11	Primary Reading Inventory Scale development	3–5 min screen to identify children at risk of reading problems (includes dyslexia) and 30-min inventory to determine reading concepts that need to be taught.	NA	Student (Can be fully administered by teacher)
12	Pupil Rating Scale Revised Screening for Learning Disabilities	5 areas of verbal and nonverbal types (auditory comprehension and memory, language, time and orientation judgment, movement and social behavior).	24 items	Teacher
13	Reading Ability Test	Two tests, one involved reading individual words and another involved reading three short passages	NA	Student (Can be administered by researcher)
14	Reading Disability Test	Subtests include vocabulary recognition, vocabulary understanding, sentence understanding and silent reading	4 subtests	Student
15	RSR-DSA derived questionnaire (Questionario per la rilevazione di difficolta‘ e disturbi dell'apprendimento)	Questions on dyslexia and closely related disorders (difficulties in math, handwriting, spelling, and reading)	34 items	Teacher
16	Screener for Reading and Writing	Instrument assessing reading and writing skills.	16 items	Teacher
17	Woodcock Johnson–Revised	Consists of letter-word identification (real words); word attack (pseudowords) and passage comprehension subtest	3 subtests	Student

NA not available.

In undertaking dyslexia screening in schools, other tools were used to assess the impact of dyslexia and or concurrent problems (Supplementary Table 2), showing how some protocols integrated cognitive and emotional measures alongside dyslexia-specific screening. We identified 23 other tools that were used, and which assessed features such as intelligence through the Wechsler Intelligence Scale for Children (WISC), emotional and behavioral screening using the Strengths and Difficulties Questionnaire, or symptoms of psychosis and mood through the Kiddie Schedule for Affective Disorders and Schizophrenia. Of these other tools, all bar one where the parent was the informant, required completion by the young person.

## Discussion

Our narrative systematic review reveals significant global differences in school-based dyslexia screening for children and young people (CYP) aged 4–16 years. The studies we included covered various countries, populations, and languages, making direct comparisons challenging. Additionally, few studies provided data on the sensitivity and specificity of the screening methods used. As a result, we were unable to determine whether school-based dyslexia screening should rely on a single tool, or a combination of tools used either together or in sequence. Our findings reinforce the need for clarity in how dyslexia is defined and operationalized in screening contexts. Tools used without consistent criteria or theoretical underpinning may contribute to under- or over-identification, especially in diverse populations.

The most commonly used dyslexia screening tool was the Developmental Dyslexia and Dysorthographia tool (second edition), which consists of eight subsets. In studies, different subsets of this tool were used for screening. It was developed based on norms from a sample of 1,200 children aged 7–14 years (33). The second most frequently used tool was the Raven Progressive Matrices, which was always combined with another screening tool. While standardized across various populations, this tool is designed to assess abstract reasoning and problem-solving abilities through pattern recognition (34), rather than specifically measuring dyslexia.

We identified several screening tools, such as the Strengths and Difficulties Questionnaire, that were not specifically designed for dyslexia screening but instead assessed the impact of dyslexia. For example, these tools measured the effects of dyslexia on an individual's psychosocial functioning, as well as emotional and behavioral difficulties. This type of supplementary screening could play an important role in school-based assessments, helping to identify children and young people with dyslexia who face the greatest challenges and guiding how resources are prioritized for them.

Research to date for dyslexia screening recommends the use of a tool with a sensitivity score of 90% (35, 36); high sensitivity scores are likely to identify nearly all children with dyslexia and that additional tests are then used to confirm an accurate diagnosis; removing the number of children identified wrongly (false positives). In our review, we found one study by Nergård-Nilssen and Friborg (32) that used a Dyslexia Marker Test that met this recommendation reporting a sensitivity of 91%. This tool consisted of six subtests: letter knowledge test, phoneme isolation, phoneme deletion, RAN, working memory, decoding and spelling and covered three areas: ability, attainment and diagnostic. The only other study conducted by Fletcher et al. (23) that nearly met this recommendation, reported a sensitivity score of 89% and used five screening tools.

The prevalence of children screening positive for dyslexia varied widely across studies, ranging from 3% to 33% of the sample population. The highest prevalence was reported in a Pakistani study (33%; 27), which used a Learning Disability Checklist. We also found that only 50% of the studies reported on the gender of students, 19% on their socio-economic status, and none on their ethnicity among those who screened positive for dyslexia. When considering educational stage, screening practices also diverged: tools for early years (ages 4–6) prioritized early phonological skills, rapid naming, and alphabet knowledge, whereas upper primary and secondary school tools often assessed fluency, comprehension, and spelling. Framing tools by school stage as well as age may help schools select the most developmentally appropriate instruments.

The wide variation in the dyslexia screening tools used in school-based research may reflect a lack of consensus on the best approach. A recent systematic review found that psychologists in English-speaking countries show little alignment in their methods for assessing dyslexia (37). Furthermore, the definition of dyslexia itself has evolved over time, with arbitrary cut-offs and the absence of clear diagnostic criteria (5, 6). This has led to differences in operational definitions and screening tools, each emphasizing different skills and characteristics (38). As a result, the lack of a unified approach to screening limits the ability to implement universal methods and complicates decisions within schools and the broader education system regarding which tool to use for dyslexia screening (38).

### Limitations

Our search strategy involved five electronic databases with a broad approach that included terms related to other neurodiverse conditions, rather than focusing solely on dyslexia-specific terms. Additionally, we did not include gray literature in our search strategy, as the focus of this review was on effective school-based dyslexia screening tools, and it is unlikely that gray literature would have provided relevant articles.

Our review was limited to dyslexia screening tools that were specifically used and studied within school settings. While this focus is context-specific, it may have excluded tools that, although not used in schools, could still be effective for school-based screening. Additionally, the wide variety of screening tools identified made it difficult to generalize the findings or make direct comparisons. The studies also involved diverse school settings and educational systems, which may limit the applicability of the findings to different schools.

Many studies also lacked complete socio-demographic data on the children and young people (CYP) being screened. This absence of information limits our ability to determine whether the screening tools are suitable for use across different socio-demographic groups. Future research should aim to include a broader range of socio-demographic factors to address these gaps.

### Strengths of review

This review has several methodological strengths. We conducted a systematic multi-database search using a protocol informed by PRISMA, with input from a young people's advisory group to ensure relevance to school settings. We also included a wide range of study designs and international settings, allowing us to highlight global variability in dyslexia screening approaches. The inclusion of both dyslexia-specific and supplementary tools (e.g., cognitive or emotional assessments) also provides a broader perspective on how schools operationalize screening in practice.

### Recommendations for policy makers

Schools vary in their financial resources, student populations, technical infrastructure, and schedules. When choosing a dyslexia screening tool, they must consider factors such as the time required to complete the screening, its sensitivity and specificity, and the potential risks of identifying a high number of false positives, children who do not actually have dyslexia. These factors can influence their decision on which screening tool to use.

Given the lack of clear evidence from our review, we propose a practical two-stage approach for school-based dyslexia screening. The first stage would involve a simple and quick screening tool, such as the Dyslexia Marker Test for Children (32), which may have a higher rate of false positives. The second stage would involve a more detailed assessment to confirm the diagnosis. However, it's important to note that more specific assessments may come with higher costs. Further research into the factors influencing decision-makers' choice of screening tools could help identify key elements that support, or hinder, the implementation of school-based screening programs. Our recommendation for a two-stage screening model is therefore pragmatic: although evidence remains limited, this approach balances feasibility (quick initial screen) with accuracy (detailed follow-up), and reflects current practice in several educational systems.

We recommend adopting universal national screening guidelines to provide a structured approach to dyslexia screening in schools, helping to prevent potential harms. Additionally, there is an urgent need for a robust evidence base on dyslexia screening in schools, with ongoing monitoring to quickly address any biases or disparities in screening uptake and identification.

## Conclusion

Various school-based screening tools are used to identify children with dyslexia, but there has been no consensus over time regarding the best approach. The wide range of dyslexia screening tools identified in this review raises questions about whether such diversity is necessary and or reflects variation in definition. Increased collaboration between researchers and front-line educators could help address these differing approaches and establish an evidence-based screening method. In the meantime, an initial screening with a highly sensitive tool, followed by more specific tests to assess detailed deficits and their impact, may provide a beneficial approach.
