# Supplementary material for: A narrative review of school-based screening tools for dyslexia among students
Source: Front Public Health. 2025 Oct 23;13:1654470. doi: 10.3389/fpubh.2025.1654470 (PMC12591040; doi:10.3389/fpubh.2025.1654470)
Supplement: Supplementary file 1 [file Data_Sheet_1.docx]

**Supplementary File 1**

POPULATION

Age:

1.pupil*.mp.

2.student*.mp.

School setting:

4.school*.mp.

5.classroom*.mp.

9. 1 or 2 or 3 or 4 or 5 or 6 or 7 or 8

INTERVENTION

10.screen*.mp. screen$2test

12. exp Mass Screening/

14. detect*.mp.

16.test*.mp.

20.tool*.mp.

24.10 or 11 or 12 or 13 or 14 or 15 or 16 or 17 or 18 or 19 or 20 or 21 or 22 or 23

Page 39

CONTROL – N/A

OUTCOME (screening result NOT diagnosis)

25. autis*.mp.

26. ASD.mp

27.Autistic disorder.m.p

28.exp Autism Spectrum Disorder/

29.Autistic Disorder/

30 attention deficit hyperactive disorder*.mp.

31.ADHD.mp.

32.exp Attention Deficit Disorder with Hyperactivity/

33.dyslexia.mp.

35.exp Dyslexia/

37.25 or 26 or 27 or 28 or 29 or 30 or 31 or 32 or 33 or 34 or 35 or 36

38. 9 AND 24 AND 37

LIMITATION 2010 onwards -to current
