# Supplementary material for: A narrative review of school-based screening tools for dyslexia among students
Source: Front Public Health. 2025 Oct 23;13:1654470. doi: 10.3389/fpubh.2025.1654470 (PMC12591040; doi:10.3389/fpubh.2025.1654470)
Supplement: Supplementary file 2 [file Table_1.docx]

**Supplementary Table 1. Eligibility criteria**

|  | **Inclusion criteria** | **Exclusion criteria** |
| --- | --- | --- |
| **Population** | School children aged 4 to 16 years | Children aged below 4 years and young people aged above 16 years. |
| **Intervention** | School-based screening tools for dyslexia by self-report, teachers, parents | Screening conducted in non-school based settings for example, healthcare, community setting. Screening tools for neurodiverse conditions other than dyslexia. Screening tools that were not self-report or used by teachers and parents. |
| **Comparison** | Primary research studies utilising dyslexia screening tools in school settings | Non-primary literature (e.g., abstracts, posters, editorials, letters); studies not involving school-based screening |
| **Outcome** | Screen positive for dyslexia, sensitivity and specificity of screening tool in a school-based setting. Prevalence of dyslexia | Screen negative for dyslexia or screen positive for any other neurodiverse or mental health condition |
| **Setting** | Any country | None |
| **Publication** | Available in English | Not available in English |
|  | Published from 2010 onwards | Published before 2010 |
