# Supplementary material for: A narrative review of school-based screening tools for dyslexia among students
Source: Front Public Health. 2025 Oct 23;13:1654470. doi: 10.3389/fpubh.2025.1654470 (PMC12591040; doi:10.3389/fpubh.2025.1654470)
Supplement: Supplementary file 3 [file Table_2.docx]

**Supplementary Table 2. Summary of other generic tools used in overall dyslexia screening included in the review (N=23).**

|  | **Screening tool** | **Description of screening tool** |
| --- | --- | --- |
| 1 | Alphabetic Knowledge | Knowledge of letter names and sounds |
| 2 | BVSCO – Battery for assessment of writing skills | Dictation test to screen reading difficulties |
| 3 | Clinical Evaluation of Language Fundamentals 3^rd^ edition: Rapid Automatic Naming, colours subtest | Evaluates general language ability through multiple subtests |
| 4 | Combined Raven Test | Combines first 3 units of Raven’s Colour Progressive Matrices & last 3 units of Raven’s Advanced Progressive Matrices |
| 5 | Comprehensive Test of Phonological Processes | 5 composite scores of phonological awareness, phonological memory, rapid symbolic, rapid non-symbolic naming, alternate phonological awareness. |
| 6 | Grade 0 oral word reading accuracy | Students attempt to read aloud 32 words |
| 7 | Grade 1 & 2 oral words reading accuracy | Students read a total of 104 words as accurately and fast as they could |
| 8 | Grade 1 & 2 oral words reading fluency | Based on the first 24 short regular words in the word reading accuracy task. |
| 9 | MT battery (Prove di lettura MT per la scuola elementare-2) | Test of reading speed and accuracy. Assesses reading abilities for meaningful texts. |
| 10 | Kiddie Schedule for Affective Disorders & Schizophrenia | Semi-structured interview measuring mood, anxiety, psychotic, and disruptive behaviour disorders |
| 11 | Paired associate learning | Visual–verbal learning task (associate nonsense word names with non-familiar cartoon animals) |
| 12 | Peabody Picture Vocabulary Test-Revised | Child hears a stimulus word, is shown 4 pictures and must choose one picture that depicts the word. |
| 13 | Phonemic awareness | 19 item to identify & delete initial, medial, or final phonemes in spoken words. |
| 14 | Rapid automatized naming | Presenting high-frequency lowercase letters repeated 10 times in random sequences |
| 15 | Raven Progressive Matrices | Test of observation & thinking |
| 16 | Slosson Intelligence Tests | Measures IQ |
| 17 | Strengths & Difficulties Questionnaire | 25 items, 5 subscales (emotional symptoms/conduct problems/hyperactivity-inattention/peer problems/lack of prosocial behaviour) |
| 18 | Strengths & Difficulties Questionnaire [Chinese] – Parent ***** | 25 items, 5 subscales (emotional symptoms/conduct problems/hyperactivity-inattention/peer problems/lack of prosocial behaviour) |
| 19 | Teacher ratings of ‘phonic phases’ | Phonic phases comprise a scale of level descriptors against which teachers assess children's phonic skills and knowledge. |
| 20 | Wechsler Intelligence Scale for Children 3^rd^ edition (WISC-III) | Block Design sub-test is a timed core perceptual reasoning sub-test. Vocabulary measures the students' verbal fluency and concept formation. |
| 21 | Word reading | Children read aloud 50 words presented |
| 22 | Working Memory Test Battery for Children | Assess digit recall forward & listening recall of memory |
| 23 | York Assessment of Reading for Comprehension (YARC) | Analysis of reading and comprehension skills. letter sound knowledge, early word reading subtest, sound deletion & isolation subtest |

***All tools have student as informant apart from * which** **has a parent informant.**
